# Alterations in epigenetic marks and expression of genes related to stress regulation: an exploratory study among newborns after fetal repair of spina bifida aperta

**DOI:** 10.1080/15592294.2026.2632976

**Published:** 2026-03-08

**Authors:** M. A. Landolt, N. L. Strebel, U. Moehrlen, N. Ochsenbein, N. Strübing, T. Burkhardt, C. D’Addario, M Pucci, A. Bodenmann, E. Grünblatt

**Affiliations:** a Department of Psychosomatics and Psychiatry, and Children’s Research Center, University Children`s Hospital Zurich, University of Zurich, Zurich, Switzerland; b Division of Child and Adolescent Health Psychology, Department of Psychology, University of Zurich, Zurich, Switzerland; c Department of Fetal Surgery, University Children’s Hospital Zurich, University of Zurich, Zurich, Switzerland; d Department of Obstetrics, University Hospital Zurich, Zurich, Switzerland; e Department of Bioscience and Technology for Food, Agriculture and Environment, University of Teramo, Teramo, Italy; f Department of Clinical Neuroscience, Karolinska Institutet, Stockholm, Sweden; g Department of Child and Adolescent Psychiatry and Psychotherapy, Psychiatric University Hospital Zurich, University of Zurich, Zurich, Switzerland; h Neuroscience Center Zurich, University of Zurich and the ETH Zurich, Zurich, Switzerland; i Zurich Center for Integrative Human Physiology, University of Zurich, Zurich, Switzerland

**Keywords:** Spina bifida aperta, NR3C1, FKBP5, epigenetics, prenatal stress, gene methylation

## Abstract

Fetal repair of spina bifida aperta (fSBA) is an established intervention that improves neurological and neurodevelopmental outcomes. The present exploratory study examines whether molecular signatures related to stress regulation are detectable in newborns following this procedure. Specifically, we investigated DNA methylation and gene expression of two stress-regulatory genes, *NR3C1* and *FKBP5*. Within a clinical trial (ID: NCT04027374), we analyzed postpartum saliva samples from newborns who had undergone fSBA repair (fSBA group; *n* = 30) and compared them with two control groups: newborns exposed to antenatal glucocorticoids for lung maturation (LMI group; *n* = 12) and healthy controls (HC group; *n* = 27). Pyrosequencing and qRT-PCR were used for epigenetic and transcriptional analyses. Significant group differences were observed in *FKBP5* methylation, particularly at intron 7 CpG sites 5–7. The fSBA group showed lower methylation at site 5 but higher methylation at sites 6–7 compared to controls. No significant methylation differences were detected for *NR3C1*. Conversely, *NR3C1* gene expression was elevated in the fSBA group, whereas *FKBP5* expression did not differ between groups. These findings suggest gene- and site-specific molecular variation in newborns following fetal surgery. Given the exploratory nature of the study, the results are not suited to draw specific clinical implications but may inform future work aimed at understanding stress-related molecular alterations surrounding fetal interventions. Larger and longitudinal studies are warranted to clarify the robustness, developmental course, and potential clinical relevance of these molecular patterns.

## Introduction

Fetal surgery has emerged as a transformative intervention for selected congenital anomalies, enabling modification of disease trajectories that would otherwise lead to severe disability or death. One such condition is spina bifida aperta (fSBA), where the spinal cord and its surrounding structures fail to close properly during early development, with its most severe form being myelomeningocele. The Management of Myelomeningocele Study (MOMS) provided compelling evidence that prenatal repair improves neurological outcomes and reduces the need for postnatal shunt placement [1]. However, as with any major medical intervention, the procedure entails a complex peri- and postoperative environment. This includes surgery-related stress such as inflammation induced by the intervention, hypoxia during anesthesia and glucocorticoid exposure, as well as additional stressors not directly caused by the surgery itself, such as maternal anxiety related to managing the high-risk pregnancy and stressors associated with the hospital environment [2–4]. While these aspects are integral components of the established clinical protocol, their associated biological signals – particularly at the molecular and epigenetic level in the fetus – remain insufficiently characterized. A better understanding of such molecular patterns, especially those related to prenatal stress, may contribute to future optimization of perioperative care and motivate further research into the biological mechanisms accompanying fetal interventions.

Epigenetic modifications, such as DNA methylation, are key mechanisms through which environmental factors, including stress, can exert long-lasting effects on gene expression. The glucocorticoid receptor gene (*NR3C1*) and the FK506 binding protein 5 gene (*FKBP5*) are of particular interest given their central roles in regulating the hypothalamic-pituitary-adrenal (HPA) axis, the body’s primary stress response system [5]. Aberrant methylation of these genes has been linked to altered stress responsivity and increased vulnerability to a range of psychiatric and neurodevelopmental disorders [6–8], consistent with the fetal programming hypothesis [9–11]. Many studies report that perinatal and maternal stress is associated with hypermethylation of *NR3C1* in offspring, whereas hypomethylation of exon 1F has been related to protective factors such as maternal-fetal attachment during pregnancy, breastfeeding, higher birth weight, or higher Apgar scores [8,12–14]. However, some studies have failed to replicate these findings [e.g. [15–17]. More recent research points to more complex and dynamic associations between adversity and DNA methylation. For example, Parent et al. [18] reported that maltreated preschool children showed higher baseline levels of *NR3C1* methylation, followed by statistically significant decreases in methylation over a six-month period and, at follow-up, lower methylation levels compared to a non-maltreated control group. This more complex view of the link between stress exposure and epigenetic modifications is also in line with what Bustamante et al. [19] reported: childhood maltreatment was associated with higher *NR3C1* methylation levels, whereas major depressive disorder was related to *NR3C1* demethylation. This also aligns with the findings reported by Na et al. [20].

Conversely, the demethylation of *FKBP5*, a key modulator of glucocorticoid receptor sensitivity, has frequently been associated with heightened stress reactivity and greater risk for anxiety and depression [8,21,22], including pronounced hypomethylation and elevated gene expression in the offspring of Holocaust survivors [23]. Nonetheless, for this gene, in addition to null-findings [21–23], some results indicating the contrary are also documented [24]. Both Monk et al. [25] and Kertes et al. [26] report hypermethylation of *FKBP5* in offspring of highly stressed mothers, blurring the picture of *FKBP5* methylation associated with early life experiences and psychopathology [27]. Child sex, developmental timing, and cell- or tissue-specificity are discussed as potential contributors to these mixed results [28,29].

These findings highlight the relevance of examining how elements of the peri- and postoperative environment surrounding fetal interventions – including physiological and procedural demands that may be stressful for the fetus or the mother – are reflected in molecular patterns of stress-regulatory genes in the operated patient. At the same time, the existing literature remains inconclusive regarding whether hyper- or hypomethylation of *NR3C1* and *FKBP5* consistently predicts later psychopathology or behavioural problems. This underscores the need for further research into the specific mechanisms involved.

### Aims and hypotheses

Fetal surgery for conditions such as fSBA provides a unique opportunity to examine epigenetic consequences of prenatal stress in a controlled clinical context. This study investigated methylation and expression levels of *NR3C1* and *FKBP5* in newborns with fSBA after fetal surgery (fSBA-group) and compared them with two cohorts: healthy newborns (HC-group) and newborns exposed to antenatal glucocorticoid treatment for lung maturation (LMI-group). The inclusion of the latter group is particularly relevant because the antenatal glucocorticoid treatment is part of the standard protocol for fetal surgery and may itself influence HPA-axis development and related epigenetic patterns [30]. By comparing these groups and simultaneously controlling for relevant co-variates including maternal stress during pregnancy, we aimed to delineate the specific effects of prenatal surgery-related stress on the epigenetic regulation of *NR3C1* and *FKBP5* and to explore how these alterations may impact gene expression. Given the heterogeneity of previous findings, we adopted an exploratory approach and refrained from formulating specific hypotheses.

The significance of this research lies in its potential to advance the understanding of how fetal interventions may be associated with molecular markers of stress regulations. Should distinct alterations in the methylation and/or expression of *NR3C1* and *FKBP5* genes be identified, such findings could contribute to ongoing efforts to optimize maternal – fetal care and refine supportive strategies before, during, and after fetal surgery. Such measures might include minimizing stress exposure during and after surgery and developing postnatal interventions aimed at mitigating the long-term consequences of these molecular changes. More broadly, the study has scientific relevance because the association between methylation of *NR3C1* and *FKBP5* and later psychopathology remains inconsistent, with both hyper- and hypomethylation reported in the literature. Given its design, the present study is well positioned to address some of these inconsistencies and to add data relevant to understanding the biological mechanisms that link early developmental environments with epigenetic variation.

## Materials and methods

### Participants

The study is registered on ClinicalTrials.gov (ID: NCT04027374) and was approved by the Cantonal Ethics Committee Zurich (approval number: 2019-00598). Patients and healthy newborns were consecutively recruited at the Department of Obstetrics of the University Hospital Zurich over a period of 42 months (June 2019 to December 2022). Inclusion criteria for the fSBA-group were a prenatally operated spina bifida aperta and a delivery via elective caesarean section (ECS) between the 35 and 37 weeks of pregnancy. The second group (LMI) included healthy newborns who underwent prenatal treatment with synthetic glucocorticoids for lung maturation due to preterm birth risk and had a delivery between the 35 and 40 weeks of pregnancy by ECS. The third group (HC) comprised healthy newborns delivered by ECS between the 36 and the 40 weeks of pregnancy. Exclusion criteria for all three groups were: (1) insufficient German/English language skills of the mother, (2) a non-Caucasian origin of the parents, (3) multiple pregnancies or pregnancy due to egg donation, (4) impaired neonatal outcome (such as 5-minutes Apgar ≤7, respiratory distress syndrome, umbilical artery pH <7.15), (5) receiving general anesthesia during cesarean section, (6) medical complications during pregnancy, and (7) trauma exposure during pregnancy. For the LMI and the HC group, premature rupture of membranes was also an excluding criterion.

According to an a-priori power analysis (effect size d = 0.4; power = .90) a minimum sample size of *N* = 84 (28 for each group) was required to conduct group comparisons using ANCOVA for our primary outcomes (methylation and expression of the *NR3C1* and *FKBP5* genes). Due to recruitment difficulties in the LMI-group, the final number of participants included in the data analyses amounted to *N* = 69 (fSBA = 30, LMI = 12, HC = 27).

### Procedure

Mothers of participating children were recruited at the hospital during weeks 30 and 35 of pregnancy. Of 117 eligible cases, 69 participated and provided written informed consent. The main reasons for non-participation were delivery or cesarean section at another hospital, premature births and related complications, medical and postoperative complications, organizational or logistical obstacles, and lack of informed consent or meeting of exclusion criteria. Infant saliva samples were collected between 24 and 72 hours postpartum by a trained research assistant. We decided against blood withdrawal in the newborns because of its invasiveness and against the use of umbilical cord blood because of documented risks of maternal contamination [31] and variability in cellular composition related to external factors such as birth weight [32]. Mothers completed paper-pencil questionnaires on psychosocial variables at 6 weeks postpartum. Medical data of mother and child were taken from the patient records.

For the saliva sample collection, the infants were refrained from eating for a minimum of 15 min before saliva collection. Samples were obtained by enabling newborns to suck or chew on the cotton swab, while a research study staff member wearing a glove held one end of a salivary swab (DNA and RNA Oragene Tubes, DNA Genotek, Canada; OC-175/OCR-100 & CP-190/ORE-100, respectively) within the infant’s buccal mucosa or sublingually to absorb saliva. The swabs were placed in the mouths of the infants for 5 min, or until the baby rejected the swab. After collection, the tube was held upright to prevent the liquid within the tube from spilling. The cap was unscrewed from the collecting tube without touching the sponge. The cap was inverted, the sponge was placed into the tube, and the cap was secured firmly. The sealed tube was inverted and shaken vigorously 15 times. DNA saliva samples were kept at room temperature and protected from light until isolation, while RNA saliva samples were stored at room temperature for up to 4 months before being transferred to −20°C for longer storage until isolation.

### Measures

Various questionnaires and a medical data sheet were used to assess the psychosocial and medical maternal and child variables. All questionnaires are standardized and available in validated English and German versions.

To assess subjective maternal distress retrospectively during pregnancy and during the last 2 weeks the Perceived Stress Scale (PSS-10 [33–35]) was administered. Using 10 items, the stress level was indicated on a 5-point Likert scale (1: never to 5: very often). Based on the manual, the sum of the items was used as an indicator of subjective stress. In this study, the PSS-10 showed a high internal consistency (Cronbach’s α of the current stress assessment = .91; Cronbach’s α of the retrospective stress assessment during pregnancy = .92).

The Patient Health Questionnaire (PHQ-9 [36]) was employed to assess the severity of maternal depressive symptoms, both retrospectively (regarding the course of the pregnancy) and currently (regarding the last 2 weeks). The self-report questionnaire consists of 10 items with a 4-point Likert scale (1: not at all to 4: almost every day) which are summed into a total score. Internal consistencies were in the satisfactory range (Cronbach’s *α* of the current mood assessment = .73; Cronbach’s *α* of the retrospective mood assessment = .77).

The occurrence of significant life events during pregnancy was assessed by means of the Life Events Scale (LES [37]) which includes 11 items with a yes/no format. For use in this study, the number of experienced life events was summed. We further assessed several medical variables related to birth and neonatal health. These included gestational age at birth, total duration of caesarean section, birth weight, birth size, and head circumference of the newborn. Additionally, Apgar scores, the umbilical artery pH values and umbilical artery lactate levels were recorded.

## Data analyses

### DNA methylation analysis

DNA isolation and cleaning procedure: 500 μL of saliva sample was purified following the prepIT®•L2P 0.5 mL manual purification procedure (DNAgenotek, Canada). For further details regarding DNA processing, see supplementary information. The EZ DNA Methylation-Lightning Kit (Zymo Research, Orange, CA, USA; D5030) was used to bisulfite convert 200–500 ng of genomic DNA to uracil for candidate gene DNA methylation analysis. The converted DNA was eluted in 12.5 μL. Based on the literature discussed in the introduction [17] and given that the current study was a pilot investigation, we focused on two genes: *NR3C1* and *FKBP5*. Pyrosequencing was used to measure DNA methylation at *NR3C1* and *FKBP5* gene CpG sites (Supporting Information Figure 1(a–b)). For further information on the pyrosequencing procedure, see supporting information. Supporting Table S1 lists the primers and genomic coordinates of the studied sequence.

**Figure 1.**
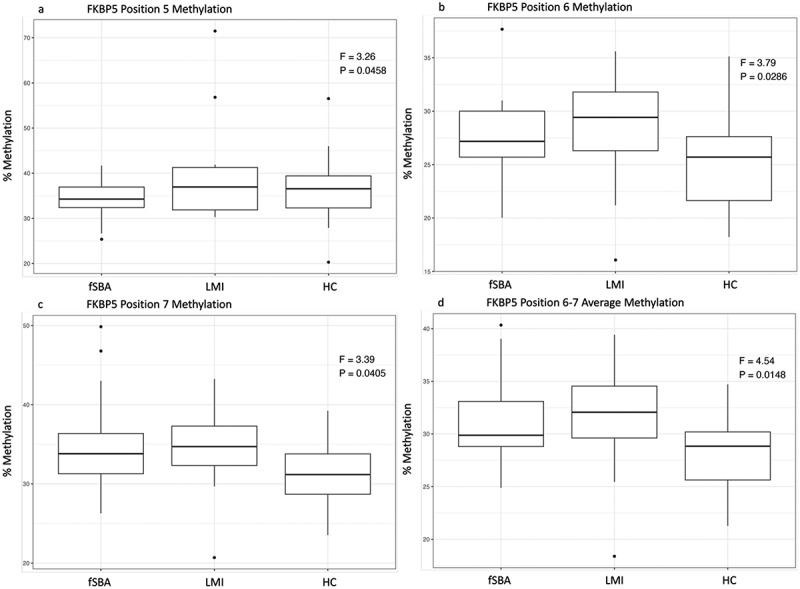
Boxplots for significant group differences in *FKBP5* DNA methylation levels (CpG site 5–7 and their average).

Note: DNA methylation percentages for intron 7 position 5–7 (a–c) and their average (d) of the *FKBP5* gene, controlled for child sex, child birthweight and child apgar2 value, are depicted as boxplots for the newborns operated for fetal spina bifida aperta (fSBA), newborns prenatally treated with synthetic glucocorticoids (LMI), and healthy controls (HC). ANCOVA F and p values are denoted, with *p* < 0.05 regarded as significant.

### RNA isolation reverse-transcription, pre-amplification, cleaning and PCR analysis

The commercial miRNeasy Micro Kit (Qiagen, Cat. No. 217,084) was used to extract RNA from RNA Oragene Tubes (DNA Genotek, CP-190/ORE-100) as described in supplementary data. 150–300 ng of total RNA were reverse transcribed into cDNA with the Whole Transcriptome Amplification Kit (WTA2, Merck/Sigma-Aldrich) and purified with NucleoSpin Gel and PCR Clean-up (740611.50; Macherey-Nagel, Switzerland). Pre-amplification with the Qiagen Multiplex PCR Plus Kit (206152) of the cDNA was conducted. Gene expression analysis of *NR3C1* and *FKBP5* was performed in triplicate (1 µL PreAmp cDNA) using QuantiNova SYBR Green detection (Qiagen, Hombrechtikon, Switzerland) and normalized with five reference genes (*RPL13a*, *PPIA*, *GAPDH*, *18S*, *ACTB*). See supporting information for method details.

### Statistical analyses

Data entry and cleansing were performed using IBM SPSS Statistics (version 28.0.0.0), while all statistical analyses were conducted in RStudio (version 2023.09.0 + 463). Two-sided tests with *p* < .05 were considered significant. Continuous variables were summarized using means, standard deviations, and ranges, while categorical variables were reported as total counts. Group differences were analyzed using ANOVAs for continuous variables and chi-squared or Fisher’s exact tests for categorical variables. Normality and multicollinearity were assessed using the Kolmogorov-Smirnov and Shapiro–Wilk tests, revealing right-skewed distributions in some cases. To achieve a normal distribution, the dependent variables (*NR3C1* and *FKBP5* expression) were log-transformed.

Separate ANCOVAs examined group differences in methylation levels at each CpG site and site-specific averages. For *FKBP5*, analyses included positions 1–7, site-specific averages (positions 1–2, 3–4, and 6–7), and the overall average for positions 1–7. For *NR3C1*, analyses included positions 1–13, site-specific averages (positions 1–5 and 6–13), and the overall average for positions 1–13. Covariates were selected based on correlation analyses with methylation levels. Five-minutes Apgar scores and birth weight were included as covariates due to significant correlations with methylation levels at three or more CpG sites. Child sex was added as a covariate given its established role in methylation processes [38,39]. Post hoc pairwise comparisons were performed using Tukey HSD. Five samples were excluded from methylation analyses due to insufficient DNA or poor pyrosequencing quality.

For gene expression analyses, separate ANCOVAs were conducted for FKBP5 and NR3C1. Relative gene expression values (CNRQ) were normalized to reference genes. A psychosocial stress sum score was computed to isolate the effects of medical stress, incorporating z-standardized means from (a) the maternal life event scale total score and (b) the mean scores of PSS-10 and PHQ-9 (both assessed retrospectively 6-week postpartum). Child sex and gestational age were included as covariates due to their documented influence on the epigenetic mechanisms [38,39]. Missing values for psychosocial stress were imputed using mean imputation to minimize data loss and distortion [40].

## Results

### Sample characteristics

Table 1 shows maternal socio-demographic and medical data across the three groups. The mothers in the fSBA-group were significantly younger than in the other two groups and less often of Swiss origin. Moreover, there were also significant differences in the mothers’ nicotine and tobacco consumption during pregnancy.

**Table 1.** Descriptive statistics for the child’s mother.

		*Group membership*						
Mother	*Total* *N* = 68	fSBA^a^ *N* = 29	LMI^b^ *N* = 12	HC^c^ *N* = 27	*F*	*p*	*χ^2^*	*p*^*d*^
Age at birth (Years), *M (SD), Range*	35.82 (4.21), 22–40	32.17^e^ (4.47), 22–40	34.67 (3.10), 29–40	35.63^e^ (3.38), 26–40	4.37	.**017**		
Gravidity (number of pregnancies) *M (SD), Range*	2.22 (1.27), 1–8	2.41 (1.24), 1–5	2.58 (1.98), 1–8	1.85 (.77), 1–4	2.03	.140		
Parity (number of births) *M (SD), Range*	1.69 (.92), 1–7	1.59 (.63), 1–3	1.83 (1.75), 1–7	1.74 (.66), 1–3	.37	.695		
Illnesses *N (%)*	61 (90)	25 (86)	11 (92)	25 (93)			.68	.712
Psychological disorder *N (%)*	5 (7)	4 (13)	0 (0)	1 (4)				.197
Medication during pregnancy *N (%)*	64 (94)	29 (100)	12 (100)	23 (85)			6.45	.168
Analgesics	10 (15)	9 (31)	1 (8)	0 (0)				.**004**
Antibiotics	40 (59)	21 (72)	6 (50)	13 (48)				.232
Dexamethasone	41 (60)	29 (100)	12 (100)	0 (0)				
Nicotine/tobacco use during pregnancy *N (%)*	4 (6)	0 (0)	3 (25)	1 (4)				.**007**
**Highest level of education**, *N (%)*	*N* = 59	*N* = 28	*N* = 11	*N* = 20			11.97	.063
University/ETH	25 (42)	9 (32)	3 (27)	13 (65)				
Highschool	19 (32)	9 (32)	7 (64)	3 (15)				
Apprenticeship (3–4 years)	14 (24)	9 (32)	1 (9)	4 (17)				
Mandatory minimum period of schooling	1 (2)	1 (4)	0 (0)	0 (0)				
**Current job situation**, *N (%)*	*N* = 58	*N* = 27	*N* = 11	*N* = 20			5.79	.215
Housewife, full-time	18 (31)	12 (44)	2 (18)	4 (19)				
Looking for work	2 (3)	0 (0)	1 (9)	1 (5)				
Employed	38 (66)	15 (56)	8 (73)	15 (76)				
**Country of birth** *N (%)*	*N* = 68	*N* = 30	*N* = 12	*N* = 26			63.46	**<.001**
Switzerland	16 (25)	1 (3)	7 (58)	8 (31)				
Germany	22 (32)	18 (60)	1 (8)	3 (12)				
Austria	6 (9)	5 (17)	0	1 (4)				
Other	24 (35)	6 (20)	4 (33)	14 (54)				

*Note*. ^a^fSBA, intervention group with fetal spina bifida aperta who received fetal surgery, values are missing for one participant; ^b^LMI, control group who received synthetic glucocorticoid for lung maturation; ^c^HC, healthy control group. ^d^*p*-values for comparisons involving at least one group with fewer than seven observations are derived using estimates from Fisher’s exact test. *p* < .05 was viewed as significant.

Table 2 shows child medical data across study groups. The data are presented in a group-specific way. The fSBA-group had the lowest gestational age at birth and the longest duration of delivery by caesarean section. The birth weight of this group was also significantly lower compared to the other groups. Group differences in birth size (cm), head circumference (cm) and Apgar (except for 10-min Apgar), umbilical artery pH, and umbilical artery lactate values were not significant. Further, the differences in stress-related variables from the child’s mother depending on group membership are displayed. The only significant difference can be seen in maternal stress during pregnancy, with LMI and fSBA indicating higher means than the healthy control group. Tukey HSD post hoc tests revealed that this difference was only significant for the comparison between LMI and HC with an estimated mean difference of 6.41 (SE = 2.29, t(66) = 2.801, *p* = 0.018), although the comparison between fSBA and HC did show marginal significance (Est = 4.16, SE = 1.75, t(66) = 2.379, *p* = 0.052).

**Table 2.** Medical data of the newborn and maternal sample characteristics of stress-related variables and group-specific presentation.

		*Group membership*					
	*Total* *N* = 61–69	fSBA^a^ *N* = 26–30	LMI^b^ *N* = 11–12	HC^c^ *N* = 24–27			
	*M (SD)*	*M (SD)*	*M (SD)*	*M (SD)*	*F*	*p*	*ɳ*^*2*^
**Medical variables of the newborns**							
Gestational age at birth (weeks)	37.36 (1.01)	37.19 (1.00)	36.73 (1.21)	37.82 (.74)	6.06	.**004**	.16
Total duration of caesarean section (min)	36.91 (17.37)	46.93 (18.11)	34.58 (13.41)	27.19 (11.66)	12.23	**<.001**	.27
Birth weight (g)	2943.68 (381.12)	2780.34 (289.64)	2851.67 (460.45)	3160.00 (334.66)	9.15	**<.001**	.22
Birth size (cm)	48.51 (2.38)	48.24 (2.54)	47.63 (2.12)	49.20 (2.19)	2.25	.114	.07
Head circumference (cm)	34.63 (1.87)	34.80 (2.19)	33.63 (1.93)	34.89 (1.30)	2.19	.120	.06
Apgar values							
1-minute Apgar	8.07 (.70)	8.08 (.74)	8.09 (.52)	8.30 (.67)	2.86	.065	.08
5-minutes Apgar	8.76 (.55)	8.62 (.56)	8.92 (.29)	8.85 (.60)	1.84	.167	.05
10-minutes Apgar	9.15 (.47)	8.97 (.33)	9.33 (.49)	9.26 (.53)	4.34	.**017**	.12
Umbilical artery pH	7.33 (.04)	7.34 (.04)	7.32 (.04)	7.33 (.05)	0.71	.498	.02
Umbilical artery lactate (mmol/l)	2.38 (1.10)	2.45 (.64)	2.25 (.59)	2.36 (1.59)	0.13	.879	.00
**Maternal stress-related variables**							
Critical life events^d^	.83 (1.09)	.79 (.96)	1.14 (1.90)	.74 (.69)	.58	.565	.02
Maternal stress during pregnancy^e^ (*retrosp.)*	16.54 (6.96)	17.8 (7.56)	20.02 (5.83)	13.61 (5.68)	4.86	.**011**	.13
Maternal stress 6 weeks postnatal^e^	14.29 (6.94)	14.86 (6.45)	15.62 (6.77)	13.05 (7.58)	.74	.480	.02
Maternal depression level during pregnancy^f^ *(retrosp.)*	6.96 (2.88)	7.00 (3.02)	7.83 (2.52)	6.53 (2.89)	.86	.426	.03
Maternal depression level 6 weeks postnatal^f^	5.50 (3.48)	5.31 (3.51)	5.72 (2.14)	5.62 (4.00)	.08	.921	.00

*Note*. ^a^fSBA, intervention group with fetal spina bifida aperta who received fetal surgery; ^b^LMI, control group who received synthetic glucocorticoid therapy for lung maturation; ^c^HC, healthy control group. ^d^Measured using the Life Event Scale [37]; ^e^Measured using the Perceived Stress Scale [34]; ^f^Measured using the Patient Health Questionnaire [36]. *p* < .05 was viewed as significant. *η*^*2*^ represents partial eta-squared, where 0.01 is considered a small effect, 0.06 a medium effect, and 0.14 a large effect.

### Gene methylation

In the fSBA group, significant changes in DNA methylation at the CpG sites 6–7 within intron seven of *FKBP5* (Table 3 and Figure 1) could be observed, with effect sizes ranging from 0.10 to 0.14, classified as medium to large. Furthermore, newborns from the fSBA group had lower *FKBP5* DNA methylation at position 5 when compared to LMI and HC. More specifically, Tukey’s HSD post hoc tests showed that the difference between fSBA and LMI was also significant with an estimated mean difference of −7.18 (SE = 2.61, t(57) = −2.755, *p* = 0.021), whilst the difference between fSBA and HC showed only marginal significance with an estimated mean difference of −5.08 (SE = 2.27, t(57) = −2.236, *p* = 0.074). At positions 6, 7, and the average of 6–7, both fSBA and LMI showed hyper-methylation when compared to HC (Figure 1). However, at position 6, only the difference between LMI and HC was significant (Est = 3.75, SE = 1.50, t(57) = 2.507, *p* = 0.039), at position 7 only the difference between fSBA and HC (Est = 3.99, SE = 1.59, t(57) = 2.509, *p* = 0.039) and for the average of 6 and 7 only the difference between fSBA and HC (Est = 3.09, SE = 1.24, t(57) = 2.483, *p* = 0.042) and LMI and HC (est = 3.83, SE = 1.49, t(57) = 2.572, *p* = 0.034) showed significance. In contrast to the *FKBP5* gene, no noteworthy differences in *NR3C1* DNA methylation were identified among the fSBA, LMI, and HC newborns (Table 4).

**Table 3.** ANCOVA for *FKBP5* intron 7 DNA methylation controlled for child sex, child birthweight and child apgar2 value.

	HC^a^		fSBA^b^		LMI^c^				
Gene Position	*M*	*SD*	*M*	*SD*	*M*	*SD*	*F*(2, 61)	p	$\eta^{2}$
FKBP5 Pos 1	38.89	5.43	39.88	3.53	38.77	4.01	0.44	0.65	0.02
FKBP5 Pos 2	49.67	3.31	49.80	3.35	49.30	4.89	0.09	0.92	0.00
FKBP5 Pos 1–2 Average	44.28	3.24	44.84	2.89	44.04	4.15	0.37	0.69	0.01
FKBP5 Pos 3	71.48	5.34	71.02	4.50	72.83	5.47	0.80	0.46	0.03
FKBP5 Pos 4	62.61	5.97	63.42	3.50	63.68	7.40	0.20	0.82	0.00
FKBP5 Pos 3–4 Average	67.04	4.59	67.22	3.39	68.25	6.36	0.41	0.67	0.01
FKBP5 Pos 5	36.19	7.19	34.17^d^	3.95	40.48^e^	12.79	3.26	**0.05**	0.10
FKBP5 Pos 6	24.98^d^	3.95	27.53	3.30	28.29^e^	5.70	3.79	**0.03**	0.12
FKBP5 Pos 7	31.05^d^	4.02	34.45^e^	5.34	34.15	5.84	3.39	**0.04**	0.11
FKBP5 Pos 6–7 Average	28.01^d^	3.47	30.99^e^	3.56	31.22^e^	5.72	4.54	**0.01**	0.14
Average FKBP5	44.98	2.72	45.75	2.42	46.79	3.65	1.82	0.17	0.06

*Note*. ^a^HC, healthy control group; ^b^fSBA, intervention group with fetal spina bifida aperta who received fetal surgery; ^c^LMI, control group who received synthetic glucocorticoid therapy for lung maturation. *p* < .05 was viewed as significant. *η*^*2*^ represents partial eta-squared, where 0.01 is considered a small effect, 0.06 a medium effect, and 0.14 a large effect. ^d,e^Subgroups with different superscripts are significantly different (*p* < .05 with Tukey HSD post hoc tests).

**Table 4.** ANCOVA for *NR3C1* exon 1F DNA methylation controlled for child sex, child birthweight and child apgar2 value.

	HC^a^		fSBA^b^		LMI^c^				
Gene Position	*M*	*SD*	*M*	*SD*	*M*	*SD*	*F*(2, 61)	p	$\eta^{2}$
NR3C1 Pos 1	1.89	0.51	1.80	0.54	1.70	0.29	0.63	0.54	0.02
NR3C1 Pos 2	2.25	0.89	1.97	0.83	2.02	1.15	0.56	0.57	0.02
NR3C1 Pos 3	1.88	1.21	1.90	2.47	1.50	0.75	0.22	0.80	0.00
NR3C1 Pos 4	1.19	0.93	1.28	0.63	0.89	0.46	0.92	0.40	0.03
NR3C1 Pos 5	1.65	0.33	1.64	0.52	1.57	0.61	0.12	0.89	0.00
NR3C1 Pos 1–5 Average	1.77	0.53	1.72	0.59	1.54	0.30	0.74	0.48	0.03
NR3C1 Pos 6	3.18	1.24	3.30	1.28	3.22	1.15	0.00	1.00	0.00
NR3C1 Pos 7	2.55	1.31	2.39	1.18	2.27	1.24	0.20	0.82	0.00
NR3C1 Pos 8	3.43	1.25	3.14	1.34	3.31	0.78	0.15	0.86	0.00
NR3C1 Pos 9	4.38	6.32	3.50	1.40	3.36	0.83	0.36	0.70	0.01
NR3C1 Pos 10	6.00	8.51	4.72	2.37	4.52	0.57	0.48	0.62	0.02
NR3C1 Pos 11	4.16	1.69	3.90	1.88	3.85	1.39	0.13	0.88	0.00
NR3C1 Pos 12	3.79	0.93	3.48	1.60	3.16	1.72	0.76	0.47	0.03
NR3C1 Pos 13	3.57	1.85	3.79	2.46	2.47	1.77	1.52	0.23	0.05
NR3C1 Pos 6–13 Average	3.88	1.48	3.53	0.89	3.27	0.66	1.25	0.29	0.04
Average NR3C1	3.07	0.88	2.83	0.65	2.60	0.44	1.68	0.20	0.06

*Note*. ^a^HC, healthy control group; ^b^fSBA, intervention group with fetal spina bifida aperta who received fetal surgery; ^c^LMI, control group who received synthetic glucocorticoid therapy for lung maturation. *p* < .05 was viewed as significant. *η*^*2*^ represents partial eta-squared, where 0.01 is considered a small effect, 0.06 a medium effect, and 0.14 a large effect.

### Gene expression

**Figure 2.**
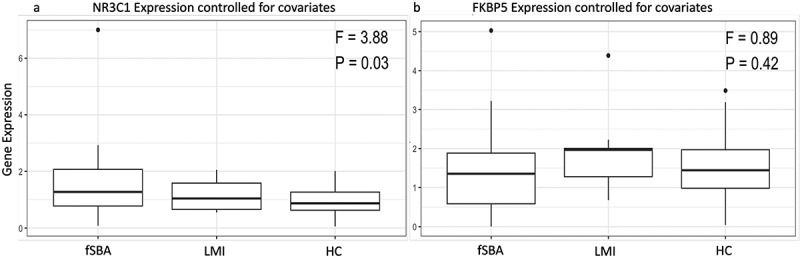
Boxplots for *NR3C1* and *FKBP5* gene expression.

Note: Boxplots for *NR3C1* and *FKBP5* gene expression, controlled for child sex, psychosocial stress and gestational age at birth. The gene expression of (a) *NR3C1* and (b) *FKBP5* in the saliva of newborns who have been operated for spina bifida aperta (fSBA), those prenatally treated with synthetic glucocorticoids (LMI), and healthy controls (HC) was evaluated using qRT-PCR. ANCOVA F and p values are denoted, with *p* < 0.05 regarded as significant.

The mRNA expression of *NR3C1* in the fSBA group was significantly elevated compared to those of LMI and HC (F = 3.82, *p* = 0.03, Figure 2(a), and supporting information in Table S2). We observed no significant changes in gene expression of *FKBP5* mRNA across the groups (Figure 2(b) and supporting information Table S2).

## Discussion

This study examined whether newborns who underwent fetal surgery for fSBA exhibit alterations in DNA methylation and gene expression in genes that are associated with stress regulation compared with two control groups: healthy newborns (HC) and newborns exposed to antenatal glucocorticoid treatment for lung maturation (LMI). Given the lack of prior research and the mixed findings reported in other prenatal stress studies, our study was exploratory, and its results should therefore be interpreted with caution.

### Gene methylation

Concerning the methylation status of genes associated with stress regulation, our findings reveal interesting differences between the *NR3C1* and the *FKPB5* gene across the three groups. Notably, we did not find any significant methylation differences between the three groups on the *NR3C1* gene while controlling for covariates. While this finding is in contrast to some previous studies that showed an association between maternal stress and higher *NR3C1* methylation [e.g. [41], it is in line with many others. For example, a recent study examining elevated maternal diurnal cortisol levels during the first trimester and depression revealed a significant association with increased DNA methylation at exon 1D of the *NR3C1* gene in the placenta, but not at exon 1F [17]. In a study of 236 pregnant women, Galbally et al. [15] found no significant differences for depression or antidepressant use in the DNA methylation of *NR3C1* in infant buccal samples within 3 days of delivery. Cecil et al. [42] investigated the cord blood of 260 individuals at birth to determine whether early-onset conduct problems are associated with prenatal environmental factors such as maternal diet, smoking, alcohol consumption, and stressful events, along with alterations in methylation; however, no changes in *NR3C1* methylation levels were observed. An even larger study by Mansell et al. [43] with 481 participants did find that maternal anxiety and psychological distress were associated with a small increase in neonate *NR3C1* methylation at specific CpG sites in the newborn. However, these findings did not remain after correction for the number of CpG sites and exposures investigated, leading them to argue for caution when interpreting previous findings. Another, smaller study by Ostlund et al. [16] failed entirely to replicate these findings, reporting no association between prenatal stress and either fearfulness or *NR3C1* methylation in 5-month-old infants, as assessed from buccal samples.

Regarding the *FKBP5* gene, this study identified CpG site-specific differences in methylation status between groups. While CpG site 5 exhibited hypomethylation in the fSBA group compared to HC, positions 6 and 7 and their average showed higher methylation levels in the fSBA group. This pattern contrasts with previous research linking higher prenatal stress exposure to *FKBP5* hypomethylation [8,17,42,44,45]. However, previous findings are mixed, and several studies have failed to replicate this association. For example, Stroud et al. [23] found no differences in *FKBP5* methylation status in placental tissue from 198 pregnant women with different trauma and PTSD histories compared to a healthy control group, despite observing higher *NR3C1* methylation in the former group. Similarly, Grasso et al. [24] found that PTSD symptoms during pregnancy correlated with higher *FKBP5* methylation in infants – a result that aligns with our findings for positions 6, 7, and the average of 6 and 7, but not for position 5. Lastly, Muller et al. [29] also failed to demonstrate a correlation between prenatal dexamethasone and perceived stress regarding children’s blood methylation levels of *FKBP5* intron 7.

The presence of comparable *FKBP5* methylation patterns in both our fSBA and LMI groups suggests that these alterations may be associated with prenatal medical and maternal stress related to complicated pregnancies, rather than being uniquely attributable to fetal spina bifida repair surgery. Accordingly, *FKBP5* methylation changes should be interpreted cautiously and not as surgery-specific molecular signatures. Overall, these mixed findings are in line with the broader literature, indicating that FKBP5 methylation is highly context-dependent and influenced by multiple biological and methodological factors, including timing of exposure, tissue type (e.g., buccal samples vs. cord blood), and developmental stage.

### Gene expression

In addition to assessing gene methylation, we also examined whether newborns who underwent prenatal surgery for treatment of fSBA show alterations in the expression of the stress-regulation genes *NR3C1* and *FKBP5* compared with two control groups. In contrast to the methylation alterations, at the gene expression level, we only observed significant changes in the expression levels of *NR3C1* in fSBA newborns. Indeed, Muller et al. [29] similarly failed to demonstrate any changes in *FKBP5* gene expression in blood samples from children of mothers treated with antenatal synthetic glucocorticoids. On the other hand, offspring mice delivered via cesarean section demonstrated increased *FKBP5* gene expression from postnatal day zero to day 56 in hippocampal samples [46]. The aforementioned study might explain the observed methylation alterations of *FKBP5* in fSBA and LMI DNA, both subjected to elevated stress levels, in contrast to the healthy control group, without identifying changes in peripheral (in our study saliva) gene expression, as the alterations may be confined to the central nervous system (in mice in hippocampus). It is important to recognize that modifications in DNA methylation at specific CpG sites do not inherently result in simultaneous changes in gene expression, as transcriptional regulation can be influenced by sequence variants, non-coding RNAs, microRNAs, and other mechanisms [47,48]. Even more so, in a study by Chatzittofis et al., NR3C1 and FKBP5 methylation levels exhibited a positive correlation with the expression of their respective genes, which is counterintuitive, as hypermethylation often suggests a suppression of gene expression [49].

Numerous studies have demonstrated that maternal stress, such as anxiety and depression, cesarean delivery, or moderate prenatal stress resulting from a superstorm event, lead to elevated gene expression of *NR3C1* in offspring [46,50,51]. In the study conducted by Muller et al. [29], prenatal dexamethasone did not affect the expression of *NR3C1* in the blood of offspring aged 7–18 years. Nonetheless, the latter may be attributed to the delayed timing of assessment, whereas all other measurements were conducted immediately at birth. Consistent with some previous findings, we detected increased *NR3C1* gene expression in fSBA offspring, which appears to diminish in a dose-dependent manner in the LMI group and further declines to a lower level in the control group.

The assumptions of a dose–response relationship should be noted as a potential explanation for the study’s findings [9,11,16,52]. The cumulative impact of medical stress is evident in the alterations of the fetal stress regulation gene *NR3C1* within the fSBA intervention group. This assumption aligns with the fetal programming hypothesis and an evolutionary-biological perspective that links a child’s adaptive performance to the degree of external stress [9,10]. This study reveals a positive correlation between increased maternal medical stress during pregnancy and newborn *NR3C1* expression (see supporting table S7), which appears to correspond to the observed dose–response effect [16,28].

## Strengths and limitations

The present study is the first to examine epigenetic alterations in genes and expression of genes that are associated with stress regulation in infants after fetal surgery in a control-group design. Moreover, saliva samples were collected in the immediate aftermath of the child’s birth, which allows to minimize postnatal influences on epigenetic alterations and gene expression. This study therefore offers a first and exploratory insight into the epigenetic consequences of fetal surgery, a method that will most probably gain importance in future years. However, there are several limitations that need to be considered when interpreting our results. First, although we had a long recruitment period, the size of the LMI-group was limited, which reduced the statistical power of group comparisons. In addition, given the exploratory nature of our study, we did not apply corrections for multiple testing to avoid overlooking potentially meaningful findings. Second, the retrospective collection of pregnancy-related distress and life events can be questioned since memory bias might have influenced the parental reports. Third, while we controlled for key variables such as child sex, birthweight or Apgar score, other potential confounding factors such as nutrition, psychosocial factors, and genetic predispositions were not assessed in this study. Moreover, even with two control groups, our design does not fully disentangle surgery-specific biological stress pathways (e.g., inflammatory or hypoxic responses) from other peri- and postoperative influences, thereby limiting conclusions regarding the specific epigenetic effects of surgery-related stress. Fourth, some results, particularly regarding *N3RC1* methylation, do not align consistently with previous findings. However, more recent literature suggests that these findings might generally not replicate and are highly tissue, time and method dependent. Lastly, due to its cross-sectional design restricted to the immediate postnatal period, this study provides only a snapshot at birth, which precludes conclusions about the stability, developmental trajectory, or functional relevance of the observed epigenetic and gene expression patterns. Longitudinal follow-up data with repeated molecular assessments and developmental outcomes would be needed to understand the long-term consequences of these modifications on childhood development, especially since epigenetic modifications are shown to be dynamic and change over time.

## Implications for research and practice

Future research should further investigate the underlying molecular mechanisms associated with prenatal surgery in individuals with neural tube defects and other major congenital malformations. Particular attention should be given to potential alterations in *NR3C1* and *FKBP5* methylation and gene expression, as well as to possible sex-specific epigenetic effects, which could not be assessed in the present study due to the limited sample size. To advance understanding of how prenatal conditions and stress may influence epigenetic programming, larger studies using genome-wide approaches, such as epigenome-wide association studies (EWAS), are warranted to identify additional biomarkers and affected pathways. Moreover, comprehensive RNA sequencing encompassing a broader array of transcripts, including genes, microRNAs, and long non-coding RNAs involved in gene regulation, may provide further valuable insights. Importantly, such research aims to expand the molecular knowledge base to optimize peri-, postoperative and long-term care of individuals that undergo prenatal surgery.

Further research should also examine placental 11β-HSD type II, an enzyme essential for regulating the transfer of maternal cortisol to the fetus. Current evidence on the association between maternal prenatal stress exposure and fetal 11β-HSD methylation remains inconsistent and inconclusive [13]. Additionally, studies investigating postnatal epigenetic alterations following critical life events in early childhood, particularly among vulnerable groups such as children with fSBA, would provide valuable insights into long-term developmental trajectories.

Beyond the dose–response relationship (cumulative hypothesis) already investigated, future research could explore the salience hypothesis, aiming to identify which psychosocial and medical stress variables are most strongly associated with epigenetic changes in the child. In this context, it would be important to explore potential thresholds at which medical stress may trigger fetal epigenetic adaptations. Moreover, examining whether even minor invasive procedures, such as amniocentesis, elicit measurable stress-related epigenetic responses could contribute to a more nuanced understanding of fetal stress regulation.

From a clinical perspective, the present findings suggest that both unborn children undergoing prenatal surgery and their mothers may be a target for preventive psychological support aimed at reducing distress in the peri- and postoperative period. It might be hypothesized that targeted stress prevention programmes could potentially attenuate alterations in the epigenome that are associated with prenatal stress exposure. Such interventions might include individual or couple-based counseling to promote emotional stabilization, as well as the mobilization of social support networks [53]. Evidence also indicates that the well-being of pregnant women can be enhanced through relaxation and guided imagery techniques, emotion regulation training, and the promotion of healthy maternal behaviours [54].

In addition to psychological approaches, stress-reducing medical strategies may also be relevant. For example, appropriate psychopharmacological treatment of maternal depression during pregnancy can contribute to greater emotional stability and may thereby support fetal development [55]. Furthermore, prenatal surgery procedures could be direct targets to minimize stress for both mother and fetus. This includes the organization of diagnostic and surgical procedures, hospital logistics and scheduling, as well as postoperative medical care to ensure a calm and supportive environment. Finally, future research should examine whether minimally invasive fetal surgery techniques may be associated with lower fetal and maternal stress levels, potentially mitigating some of the epigenetic alterations observed in this study.

## Conclusion

This exploratory study provides initial evidence that fetal surgery for spina bifida aperta is associated with gene- and site-specific alterations in stress-related molecular markers at birth. While no consistent differences in *NR3C1* DNA methylation were observed, distinct *FKBP5* intron 7 methylation patterns and increased NR3C1 gene expression in the fSBA group suggest differential regulation of components of the HPA-axis–related stress system. These findings highlight the complexity of epigenetic responses to prenatal medical stress and argue against uniform interpretations of methylation changes. Given the exploratory design and limited sample size, larger longitudinal studies are required to clarify the robustness and developmental relevance of these molecular patterns.

## Supplementary Material

Supplemental Material

## Data Availability

The data of this study are not publicly available due to their sensitive nature. They can be obtained from the corresponding author upon reasonable request and with approval from the ethics committee. All data are stored in a controlled-access repository at the University Children’s Hospital Zurich.
