# Supplementary material for: Alterations in epigenetic marks and expression of genes related to stress regulation: an exploratory study among newborns after fetal repair of spina bifida aperta
Source: Epigenetics. 2026 Mar 8;21(1):2632976. doi: 10.1080/15592294.2026.2632976 (PMC12973468; doi:10.1080/15592294.2026.2632976)
Supplement: Supplemental Material [file KEPI_A_2632976_SM2069.docx]

**Supporting Information for**

**Alterations in epigenetic marks and expression of genes related to stress regulation: an exploratory study among newborns after fetal repair of spina bifida aperta**

Landolt, M. A., Strebel, N. L., Moehrlen, U., Ochsenbein, N., Strübing, N., Burkhardt, T., D’Addrio C., Pucci M, Bodenmann, A., & Grünblatt, E

Markus Andreas Landolt

E-Mail: [markus.landolt@kispi.uzh.ch](mailto:markus.landolt@kispi.uzh.ch)

This file includes:

Supporting text

Fig. S1

Tables S1 to S7

SI References

**Supporting Information Text**

**DNA methylation analysis**

Following DNA isolation from saliva samples, the DNA pellet was dissolved with 50 µL of TE buffer. The concentrations and purity of DNA were assessed using the NanoDrop™ One Spectrophotometer (Thermo Fisher Scientific, Wilmington, DE, USA). The DNA samples were purified and concentrated according to the Genomic DNA Clean & Concentrator-10 kit procedure with minor modifications (D4010; Zymo Research, USA). DNA was washed with 14 µL water instead of 10 µL in order to increase the volume for the methylation step.

A biotin- labeled primer and the PyroMark PCR Kit (Qiagen, Hilden, Germany) amplified bisulfite-treated DNA (3–15 ng) as directed by the manufacturer. Heat at 95 °C for 15 minutes, then 45 cycles of 94 °C for 30 seconds, 51.8 °C for 30 seconds, and 72 °C for 30 seconds, concluding with 72 °C for 10 minutes [1, 2]. Electrophoresis confirmed PCR product specificity. PyroMark Q48 Autoprep with Pyro Mark Gold reagents (Qiagen, Hilden, Germany) sequenced [3]. The positive and negative methylation controls were three Qiagen-purchased standard human DNA samples: fully methylated and bisulfite converted, unmethylated and bisulfite converted, and unmethylated DNA. The PyroMark Q48 Autoprep version 2.4.2 software calculated the methylation percentage as mC/(mC + C) for each CpG site, facilitating quantitative comparisons.

| **Gene** | **CpG sites covered by amplicon** | **Amplicon size (bp)** | **CpG detected by sequencing primer** | **Primer name** | **Primer sequence** |
| --- | --- | --- | --- | --- | --- |
| ***FKBP5*** | 1,2 | 109 |  | FKBP5int7_fwd01 | TTGGAGAAGTATAAAAAAAAAATGGT |
|  |  |  |  | FKBP5int7_rev0_[BIO | [BIO]CAAATTATTATCCCAACTACAAATCCT |
|  |  |  | 1,2 | FKBP5int7_seq01 | AGAAGTATAAAAAAAAAATGGTTT |
|  | 3,4,5 | 199 |  | FKBP5int7_fwd07 | GGATAATAATTTGGAGTTATAGTGTAGGT |
|  |  |  |  | P13 FKBP5int7_1.2_R | [BIO]AAAATCCAAAACTTATTCCC |
|  |  |  | 3,4 | FKBP5int7_seq03 | GAGTTATAGTGTAGGTTTTTT |
|  |  |  | 5 | FKBP5int7_seq06 | TTTAAGGAGTTATTTGGTAGA |
|  | 6,7 | 163 |  | FKBP5int7_fwd03 | AGGGAATAAGTTTTGGATTTTATTTAAA |
|  |  |  |  | FKBP5int7_rev0_[BIO] | [BIO]TCAAATTTATCTCTTACCTCCAACACT |
|  |  |  | 6,7 | FKBP5int7_seq08 | GATATATAGGAATAAAATAAGAATA |
| ***NR3C1*** | 1-13 | 162 |  | NR3C1pro1_fwd | AGTTTTAGAGTGGGTTTGGAG |
|  |  |  |  | NR3C1pro1_rev_[BIO] | [BIO]CCCCCAACTCCCCAAAAA |
|  |  |  | 1-5 | NR3C1pro1_S1 | GAGTGGGTTTGGAGT |
|  |  |  | 6-13 | NR3C1pro1_S2 | GTAGAGAGAAAAGAAATTGGAGA |

**Table S1.** Pyrosequencing primer information

**RNA Isolation reverse-transcription, pre-amplification, cleaning and PCR analysis**

The extraction of RNA from RNA Oragene Tubes (DNA Genotek, CP-190/ORE-100) was conducted with the commercially available miRNeasy Micro Kit (Qiagen, Cat. No. 217084) as follows: The saliva samples were vigorously shaken for a minimum of 8 seconds, followed by incubation at 50°C for 2 hours. Subsequently, 400-500 µL of saliva sample was transferred to a 2 mL tube and incubated at 90°C for 15 minutes, followed by cooling to ambient temperature. 1 mL of TRI reagent was added and vortexed for 1 minute. The tube was maintained at room temperature for 5 minutes, after which 100 µL of 1-Bromo-3-chloropropane was added and vortexed for 15 seconds. The homogenate was incubated at room temperature for 2-3 minutes, then subjected to centrifugation at 12,000 x g for 15 minutes. 600 µL of the upper aqueous phase, containing the RNA, was transferred into a 2 mL tube, and 900 µL of 100% ethanol was added and mixed well by pipetting up and down. Subsequently, up to 700 µL of the sample was placed into a RNeasy MiniElute spin column, accompanied by a 2 mL collecting tube. The centrifugation was conducted at ≥8000 x g for 15 seconds at ambient temperature, and the supernatant was discarded. The centrifugation step was repeated using the remaining sample from the previous step. Subsequently, 700 μL of Buffer RWT was added to the RNeasy MinElute spin column, followed by centrifugation for 15 seconds at ≥8000 x g to wash the column, with the flow-through being discarded. 500 μL of Buffer RPE was added to the RNeasy MinElute spin column and centrifuged for 15 seconds at ≥8000 x g to wash the column. Subsequently, an additional 500 μL of Buffer RPE was introduced to the RNeasy MinElute spin column, which was then centrifuged for 2 minutes at ≥8000 x g to dry the membrane of the column. The RNeasy MinElute spin column was thereafter placed into a fresh 1.5 ml collection tube, and 25 μL of RNase-free water was added directly onto the membrane of the column. The column was then centrifuged for 1 minute at ≥8000 x g to elute the RNA. The final step was repeated to maximize the total RNA yield. The purity and quantity of total RNA were assessed spectrophotometrically using the NanoDrop One (Thermo Scientific). 150-300 ng of total RNA were reverse transcribed into cDNA with the Whole Transcriptome Amplification Kit (WTA2, Merck/Sigma-Aldrich) followed by cDNA purification using the NucleoSpin Gel and PCR Clean-up (740611.50; Macherey-Nagel, Switzerland). The cDNA underwent pre-amplification with the Multiplex PCR Plus Kit from Qiagen (206152). All QuantiTect Primer Assays were diluted to 1 μM with TE and utilized as a primer mix in the PCR, employing 50 ng of cDNA per reaction. Gene expression analysis of *NR3C1* and *FKBP5* was conducted in triplicate (1 µL PreAmp cDNA) utilizing the QuantiNova SYBR Green detection method (Qiagen, Hombrechtikon, Switzerland). This quantitative real-time reverse-transcription polymerase chain reaction employed specific detection primers for *NR3C1* (QT00020608; melting 77.5^o^C) and *FKBP5* (QT00056714, melting 79.5^o^C), alongside five reference genes—*RPL13a* (QT00089915, melting 85^o^C), *PPIA* (QT00052311, melting 81^o^C), and *GAPDH* (QT00079247, melting 81^o^C), *18S* (QT00199367, melting 85^o^C), *ACTB* (QT00095431, melting 85.5^o^C)—for normalization (Qiagen). The specificity of PCR was validated using melting curve measurements for each triple. Gene expression and normalization analysis was performed utilizing the qBase plus software (Biogazelle, The Netherlands), which employs a specialized algorithm derived from geNORM [4] to consolidate the most stable reference genes into a normalization value (*GAPDH*, *RPL13a* and *PPIA*). Additionally, PCR efficiency was assessed using the LinRegPCR software ([www.hartfaalcentru.nl](https://www.hartfaalcentru.nl)).

| ***Controlled for sex*** | fSBA^a^  *N* = 30 | |  | LRI^b^  *N* = 12 | |  | HC^c^  *N* = 27 | | F | *p* | ɳ^2^ |
| --- | --- | --- | --- | --- | --- | --- | --- | --- | --- | --- | --- |
|  | *M* | *SD* |  | *M* | *SD* |  | *M* | *SD* |  |  |  |
| FKBP5 (Expression) | 1.41 | 1.13 |  | 1.87 | .97 |  | 1.48 | .88 | .87 | .424 | .03 |
| NR3C1 (Expression) | 1.62^d^ | 1.29 |  | 1.15 | .57 |  | .92^e^ | .42 | 4.11 | .021 | .12 |
| ***Controlled for psychosocial stress*** | fSBA^a^  *N* = 30 | |  | LRI^b^  *N* = 12 | |  | HC^c^  *N* = 27 | | F | *p* | ɳ^2^ |
|  | *M* | *SD* |  | *M* | *SD* |  | *M* | *SD* |  |  |  |
| FKBP5 (Expression) | 1.41 | 1.13 |  | 1.87 | .97 |  | 1.48 | .88 | .87 | .425 | .03 |
| NR3C1 (Expression) | 1.62^d^ | 1.29 |  | 1.15 | .57 |  | .92^e^ | .42 | 4.08 | .022 | .11 |
| ***Controlled for gestational age at birth^c^*** | fSBA^a^  *N* = 30 | |  | LRI^b^  *N* = 12 | |  | HC^c^  *N* = 27 | | F | *p* | ɳ^2^ |
|  | *M* | *SD* |  | *M* | *SD* |  | *M* | *SD* |  |  |  |
| FKBP5 (Expression) | 1.41 | 1.13 |  | 1.87 | .97 |  | 1.48 | .88 | 0.86 | .428 | .03 |
| NR3C1 (Expression) | 1.62^d^ | 1.29 |  | 1.15 | .57 |  | .92^e^ | .42 | 3.81 | .028 | .11 |
| ***Controlled for all three covariates*** | fSBA^a^  *N* = 30 | |  | LRI^b^  *N* = 12 | |  | HC^c^  *N* = 27 | | F | *p* | ɳ^2^ |
|  | *M* | *SD* |  | *M* | *SD* |  | *M* | *SD* |  |  |  |
| FKBP5 (Expression) | 1.41 | 1.13 |  | 1.87 | .97 |  | 1.48 | .88 | 0.89 | .417 | .03 |
| NR3C1 (Expression) | 1.62^d^ | 1.29 |  | 1.15 | .57 |  | .92^e^ | .42 | 3.88 | .026 | .12 |

**Table S2.** ANCOVA for group comparisons (fSBA, LRI, HC) regarding epigenetic changes in the infant, controlled for sex, psychosocial stress, and gestational age at birth.

*Notes. ^a^fSBA, intervention group with myelomeningocele who received fetal surgery; ^b^LRI, control group who received synthetic glucocorticoid therapy for maturation of lung function; ^c^HC, healthy control group. Subgroups with different superscripts are significantly different (p < .05 with Tukey HSD post hoc tests).*

**Table S3.** Tukey HSD tests for *FKBP5* Pos. 5

| **Contrast** | **Estimate** | **SE** | **df** | **t ratio** | **p value** |
| --- | --- | --- | --- | --- | --- |
| **fSBA - LRI** | -7.18 | 2.61 | 57 | -2.755 | 0.02 |
| **fSBA - HC** | -5.08 | 2.27 | 57 | -2.236 | 0.07 |
| **LRI - HC** | 2.1 | 2.72 | 57 | 0.771 | 0.72 |

**Table S4.** Tukey HSD tests for *FKBP5* Pos. 6

| **Contrast** | **Estimate** | **SE** | **df** | **t ratio** | **p value** |
| --- | --- | --- | --- | --- | --- |
| **fSBA - LRI** | -1.56 | 1.43 | 57 | -1.091 | 0.52 |
| **fSBA - HC** | 2.19 | 1.25 | 57 | 1.754 | 0.20 |
| **LRI - HC** | 3.75 | 1.5 | 57 | 2.507 | 0.04 |

**Table S5.** Tukey HSD tests for *FKBP5* Pos. 6

| **Contrast** | **Estimate** | **SE** | **df** | **t ratio** | **p value** |
| --- | --- | --- | --- | --- | --- |
| **fSBA - LRI** | 0.07 | 1.82 | 57 | 0.04 | 0.99 |
| **fSBA - HC** | 3.99 | 1.59 | 57 | 2.509 | 0.04 |
| **LRI - HC** | 3.91 | 1.9 | 57 | 2.055 | 0.11 |

**Table S6.** Tukey HSD tests for *FKBP5* Pos. 6-7 average

| **Contrast** | **Estimate** | **SE** | **df** | **t ratio** | **p value** |
| --- | --- | --- | --- | --- | --- |
| **fSBA - LRI** | -0.75 | 1.43 | 57 | -0.522 | 0.86 |
| **fSBA - HC** | 3.09 | 1.24 | 57 | 2.483 | 0.04 |
| **LRI - HC** | 3.83 | 1.49 | 57 | 2.572 | 0.03 |

**Table S7.** Correlation between possible covariates and expression of *NR3C1* and *FKBP5 genes*.

| Covariates | *NR3C1* Expression | | *FKBP5* Expression | |
| --- | --- | --- | --- | --- |
|  | r | p | r | p |
| Psychosocial stress during pregnancy | 0.10 | 0.42 | -0.16 | 0.20 |
| PHQ | 0.03 | 0.83 | 0.00 | 0.98 |
| PSS | 0.09 | 0.45 | -0.24 | 0.05 |
| LES | 0.05 | 0.71 | -0.05 | 0.71 |
| Medical stress during pregnancy | 0.26 | 0.03 | -0.14 | 0.25 |
| Child gestation age | -0.23 | 0.07 | -0.17 | 0.19 |
| Hospitalization duration Mother | 0.29 | 0.02 | -0.05 | 0.71 |
| Duration of caesarean section | 0.14 | 0.25 | -0.20 | 0.11 |
| Child birthweight | -0.19 | 0.12 | -0.05 | 0.68 |
| APGAR 2 | -0.17 | 0.17 | 0.03 | 0.83 |
| Child lactate value | -0.01 | 0.95 | -0.30 | 0.02 |
| Duration of operation on child | -0.01 | 0.97 | -0.13 | 0.51 |
| Time difference between birth and saliva probe | 0.04 | 0.77 | -0.04 | 0.77 |


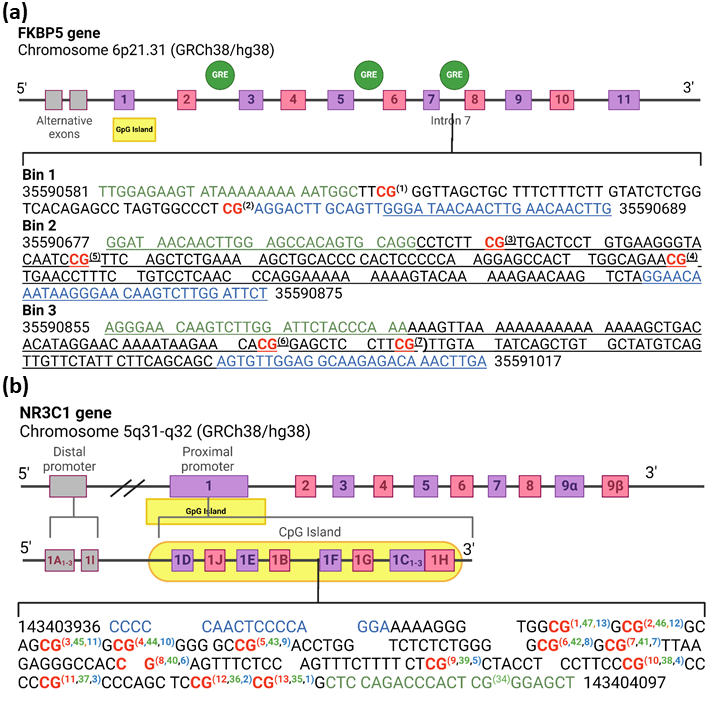


**Fig. S1.** Schematic representation of the DNA methylation of the *FKBP5* locus (a) and of *NR3C1* locus (b).

**Notes.** The locations of the sequences analyzed in this study are represented, with the sequences of the forward (**green**) and reverse primers (**blue**). (a) The CpG locations (**red**) at the intron 7 and glucocorticoid response elements (GRE; underline represents the regulatory region) are shown; CpGs are numbered according to Klengel, Mehta (5) including Bin 3 from Farrel et al. 2018. (b) The CpG locations (**red**) at the proximal promoter and exon 1 where a CpG island exists are shown; the current CpGs are numbered in **red**, in **green** the numbers are as published in Palma-Gudiel, Cordova-Palomera (6), while in **blue** the numbers represent those published in Paquette, Lester (7).

**SI References**

1. Di Bartolomeo M, Stark T, Di Martino S, et al. The Effects of Peripubertal THC Exposure in Neurodevelopmental Rat Models of Psychopathology. Int J Mol Sci. 2023;24(4):3907. doi: 10.3390/ijms24043907

2. Nassan M, Veldic M, Winham S, et al. Methylation of Brain Derived Neurotrophic Factor (BDNF) Val66Met CpG site is associated with early onset bipolar disorder. J Affect Disord. 2020;267:96-102. doi: 10.1016/j.jad.2020.02.002

3. D’Addario C, Pucci M, Bellia F, et al. Regulation of oxytocin receptor gene expression in obsessive–compulsive disorder: a possible role for the microbiota-host epigenetic axis. Clin Epigenetics. 2022;14(1). doi: 10.1186/s13148-022-01264-0

4. Vandesompele J, De Preter K, Pattyn F, et al. Accurate normalization of real-time quantitative RT-PCR data by geometric averaging of multiple internal control genes. Genome Biol. 2002;3(7):research0034.1. doi: 10.1186/gb-2002-3-7-research0034

5. Klengel T, Mehta D, Anacker C, et al. Allele-specific FKBP5 DNA demethylation mediates gene-childhood trauma interactions. Nat Neurosci. 2013;16(1):33-41. doi: 10.1038/nn.3275

6. Palma-Gudiel H, Cordova-Palomera A, Eixarch E, et al. Maternal psychosocial stress during pregnancy alters the epigenetic signature of the glucocorticoid receptor gene promoter in their offspring: a meta-analysis. Epigenetics. 2015;10(10):893-902. doi: 10.1080/15592294.2015.1088630

7. Paquette AG, Lester BM, Lesseur C, et al. Placental epigenetic patterning of glucocorticoid response genes is associated with infant neurodevelopment. Epigenomics. 2015;7(5):767-79. doi: 10.2217/epi.15.28
